# Glypican-2 levels in cerebrospinal fluid predict the status of adult hippocampal neurogenesis

**DOI:** 10.1038/srep46543

**Published:** 2017-04-25

**Authors:** S. Lugert, T. Kremer, R. Jagasia, A. Herrmann, S. Aigner, C. Giachino, I. Mendez-David, A. M. Gardier, J. P. Carralot, H. Meistermann, A. Augustin, M. D. Saxe, J. Lamerz, G. Duran-Pacheco, A. Ducret, V. Taylor, D. J. David, C. Czech

**Affiliations:** 1 Roche Pharmaceutical Research and Early Development, NORD Discovery & Translational Area, Roche Innovation Center Basel, F. Hoffmann-La Roche Ltd, Grenzacherstrasse 124, 4070 Basel, Switzerland; 2 Roche Pharmaceutical Research and Early Development, Pharmaceutical Sciences, Roche Innovation Center Basel, F. Hoffmann-La Roche Ltd, Grenzacherstrasse 124, 4070 Basel, Switzerland; 3 Embryology and Stem Cell Biology, Department of Biomedicine, University of Basel, Mattenstrasse 28, CH-4058 Basel, Switzerland; 4 CESP/UMR-S 1178, Univ. Paris-Sud, Fac. Pharmacie, INSERM, Université Paris-Saclay, Chatenay Malabry, 92290, France; 5 Roche Pharmaceutical Research and Early Development, Therapeutic Modalities, Roche Innovation Center Basel, F. Hoffmann-La Roche Ltd, Grenzacherstrasse 124, 4070 Basel, Switzerland

## Abstract

Adult hippocampal neurogenesis is a remarkable form of brain plasticity through which new neurons are generated throughout life. Despite its important roles in cognition and emotion and its modulation in various preclinical disease models, the functional importance of adult hippocampal neurogenesis in human health has not been revealed because of a lack of tools for monitoring adult neurogenesis *in vivo*. Therefore, we performed an unbiased proteomics screen to identify novel proteins expressed during neuronal differentiation using a human neural stem cell model, and we identified the proteoglycan Glypican-2 (Gpc2) as a putative secreted marker of immature neurons. Exogenous Gpc2 binds to FGF2 and inhibits FGF2-induced neural progenitor cell proliferation. Gpc2 is enriched in neurogenic regions of the adult brain. Its expression is increased by physiological stimuli that increase hippocampal neurogenesis and decreased in transgenic models in which neurogenesis is selectively ablated. Changes in neurogenesis also result in changes in Gpc2 protein level in cerebrospinal fluid (CSF). Gpc2 is detectable in adult human CSF, and first pilot experiments with a longitudinal cohort indicate a decrease over time. Thus, Gpc2 may serve as a potential marker to monitor adult neurogenesis in both animal and human physiology and disease, warranting future studies.

Adult hippocampal neurogenesis occurs in mammals, from mice to humans^1^,^2^,^3^. Almost 2% of the dentate granule cell population is renewed per year in adults^1^. A plethora of animal studies have implicated adult-born hippocampal neurons, which have different physiological properties than mature neurons, in many non-mutually exclusive roles in brain function, such as formation, spatial memory, pattern separation and the regulation of stress and affective states. Furthermore, in animal models of neurodevelopmental disorders, Alzheimer’s disease, stress and depression, alterations in neurogenesis that correlate with behavioral and cognitive symptoms have been observed and are modulated with pro- or anti-neurogenic stimuli^4^,^5^. Ultimately, the ability to track neurogenesis in longitudinal studies of living human brains will allow researchers to establish the importance of this process in physiology and disease, ultimately impacting the diagnosis and therapy of CNS disorders. A metabolic peak was identified as a potential biomarker for neurogenesis using proton magnetic resonance spectroscopy (hMRS)^6^,^7^. However, uncertainty remains regarding the specificity of the peak for neurogenesis, the spectral processing method used and the reproducibility of these results. Thus, a robust biomarker for neurogenesis remains elusive. Neurogenesis-specific biomarkers in CSF would serve as proxies for the rates of neurogenesis in the brain.

We performed an unbiased discovery proteomics screen in a stem cell model of human brain development to identify potential CSF biomarkers of neuronal differentiation^8^. The expression pattern of the membrane-bound and secreted Glypican-2 (Gpc2) protein correlated with the patterns of neurogenesis markers in immature neurons, including Dcx, Gpr56 and Dpyl4. Glypicans belong to the conserved protein family of heparan sulfate proteoglycans (HSPGs) that are characterized by a core protein with one or more covalently attached heparan sulfate (HS) chains, a type of glycosaminoglycan (GAG)^9^.

Six family members, Gpc1-Gpc6, are all expressed in the developing nervous system, but only Gpc2 expression is exclusively restricted to the CNS^9^,^10^. HSPGs have been shown to play an important role in CNS development as regulators of signaling pathways and synaptic organizers^11^,^12^,^13^,^14^. However, a functional role for Gpcs in the context of adult neurogenesis has not yet been described.

Using immunocompetitive capture coupled mass spectrometry, we showed that Gpc2 binds to Fibroblast Growth Factor 2 (FGF2) and its receptor. Gpc2 modulates neural progenitor cell proliferation by antagonizing the mitotic effect of FGF2. In rodents, Gpc2 expression is restricted to the neurogenic regions, the subgranular zone (SGZ) of the dentate gyrus (DG) and the subventricular zone-rostral migratory stream-olfactory bulb region (SVZ/RMS/OB), and can be detected in rodent and human CSF. We showed a marked reduction of Gpc2 in the CSF after genetic ablation of adult neurogenesis, thus addressing its specificity. In addition, known physiological modulators of adult neurogenesis, including running wheel and fluoxetine treatments, led to increased Gpc2 levels in CSF; conversely, aging led to a marked reduction in Gpc2 levels. Thus, CSF Gpc2 levels reflect the degree of adult hippocampal neurogenesis. Gpc2 was also detected in human CSF, and the initial experiments showed a moderate age-dependent reduction in Gpc2 levels in a small longitudinal cohort. Based on these results, Gpc2 may be a potential translational biomarker, justifying further investigations of adult human hippocampal neurogenesis and neurogenic activity in response to drug treatments.

## Results

### Proteomic profiling of differentiating human NPCs identifies Glypican-2 as a novel protein marker of neuronal differentiation

**Figure 1:**
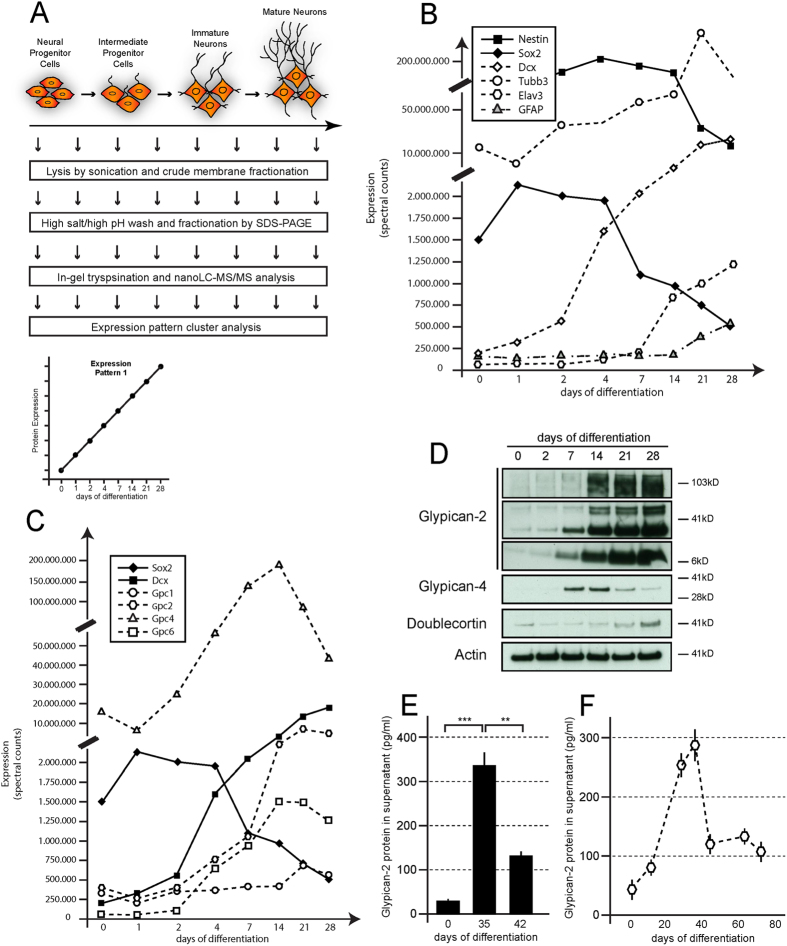
Proteomic phenotyping of differentiating NPCs identifies Glypican-2 as a marker of neuronal differentiation. (**A**) Scheme of the experimental design and work flow. Samples for proteomic phenotyping were collected at various stages during neuronal differentiation and subsequently analyzed by discovery mass spectrometry. (**B**) The expression of neural progenitor markers (Nestin and Sox2) decreases during neural differentiation, whereas the expression of neuronal marker proteins (Dcx, Tubb3 and Elav3) subsequently and sequentially increases. At late stages, proteins associated with glial identity (GFAP) are detected. (**C**) Members of the Glypican protein family (Gpc1, 2, 4 and 6) show distinct expression patterns, with a moderate to strong increase in expression during neuronal differentiation. The Gpc2 expression pattern is similar to that of Dcx. (**D**) Biochemical analysis of lysates derived from human neural progenitor cells during differentiation for Gpc2, Gpc4, Dcx and Actin expression during neuronal differentiation. (**E,F)** Analysis of soluble Gpc2 in the tissue culture supernatant by immunoassay. Gpc2 is released into the supernatant, and Gpc2 expression is transiently regulated during neuronal differentiation. Expression peaks after 5 weeks of differentiation. Error bars represent the SEM. p < 0.05 = *; p < 0.01 = **; p < 0.001 = ***.

We performed a discovery proteomics screen of human embryonic stem cell-derived neural progenitor cells (hNPCs) at different developmental stages after the onset of neuronal differentiation to identify novel protein markers of neuronal differentiation. Protein lysates were collected at different days after the induction of neural differentiation and subjected to mass spectrometry analysis (Fig. 1a). Overall approximately 4,400 proteins were dynamically expressed during the differentiation process (Fig. 1 and Supp. Fig. 1). Known stage-specific markers of neurogenesis and markers of adult hippocampal neurogenesis were dynamically regulated. The expression of neural progenitor markers, such as Sox2 and Nestin, decreased as the cells matured, whereas the expression of markers of immature neurons, including Dcx, NCAM1, Gpr56, Tubb3 and Elav3, increased over time (Fig. 1b and Supp. Fig. 1)^15^. Moreover, we detected the expression of glial markers, including GFAP and S100β (Fig. 1b and Supp. Fig. 1), at the latest maturation stages analyzed, indicating that gliogenesis followed a wave of neurogenesis, similar to physiological CNS development. Based on these data, we concluded that key elements of neurodevelopment were recapitulated using this NPC-based culture system^8^.

We hypothesized that proteins displaying a dynamic expression pattern similar to known stage-specific marker proteins might be novel markers of immature neurons. Thus, we clustered the proteins according to their dynamic expression patterns during neural differentiation (Supp. Fig. 1). Because individual peptide fragments were aligned to various isoforms of a given protein, some proteins, such as Dcx and NCAM1, fell into more than one category (Supp. Fig. 1). The proteins were evenly distributed among the categories when we analyzed general changes, such as increased, decreased and unaltered expression. Approximately one-third of the proteins displayed an increase in expression during differentiation (Expression Patterns 1–3, 1,757 proteins), another third showed a decrease in expression (Expression Patterns 7–9, 1,500 proteins) and the last third was unaltered between the NPC state (d0) and neuronal state (d28), although differential expression was observed between d0 and d28 (Expression Patterns 4–6 and 10–12) (Supp. Fig. 1). Established reference proteins for neural differentiation and markers of immature neurons, such as Dcx, Gpr56, DPYL4, NCAM1 and Elav3, were mainly included in Pattern 1. Hence, we reasoned that some of the proteins within Expression Pattern 1 may represent novel marker proteins relevant to adult neurogenesis. This assumption was supported when the transcript expression of these proteins was referenced to the Allen Brain Atlas (http://mouse.brain-map.org)^16^. Many proteins were expressed in the dentate gyrus, and several, including Glypican-2, Gpr56 and PDE8b, were enriched in or restricted to the SGZ. We focused our efforts on the Glypicans (Gpc) because (i) several members were dynamically expressed, (ii) Gpc6 and Gpc2 were among the top 50 proteins within Expression Pattern 1 and (iii) Gpcs are subject to proteolytic cleavage and could thus potentially serve as CSF biomarkers for neurogenesis.

Gpcs are membrane-bound proteins belonging to the family of heparan sulfate proteoglycans (HSPGs)^17^ and can be released into the extracellular matrix (ECM). These proteins modulate the availability of ligands for major signaling pathways and have recently been implicated as playing an important role in synapse formation^13^,^14^. Thus, Gpcs are potentially important factors within the stem cell niche that regulate the proliferation and differentiation of newborn precursor cells. We analyzed expression of the Gpc2 and Gpc4 proteins in whole cell lysates obtained from differentiating NPCs using standard biochemical methods to corroborate the results obtained in the phenotypic proteomic screen (Fig. 1d). Gpc4 expression peaked 2 weeks after the induction of differentiation, whereas Gpc2 expression showed a gradual increase over the analyzed period of 4 weeks. Furthermore, Gpc2 showed a similar expression pattern compared to Dcx, indicating a similar temporal pattern of expression. As Gpc2 is the only member of the Glypican family that is exclusively expressed in the central nervous system, we focused our subsequent analyses on this protein^10^.

Gpcs are attached to the plasma membrane via a GPI anchor but are proteolytically cleaved and released into the ECM^18^,^19^. We specifically examined the release of Gpc2 protein into the cell culture supernatant (Fig. 1e) and prolonged the period of differentiation. The amount of the released protein in the supernatant peaked 35 days after the induction of differentiation and decreased thereafter to reach a steady state level after approximately 42 days (Fig. 1e,f).

Thus, Gpc2 is dynamically and transiently expressed during the neural differentiation of human neural stem cells. It is released into the extracellular milieu, suggesting that Gpc2 has the potential to function as a CSF marker of neurogenesis.

### Glypican-2 binds FGF2 and modulates progenitor cell proliferation

**Figure 2:**
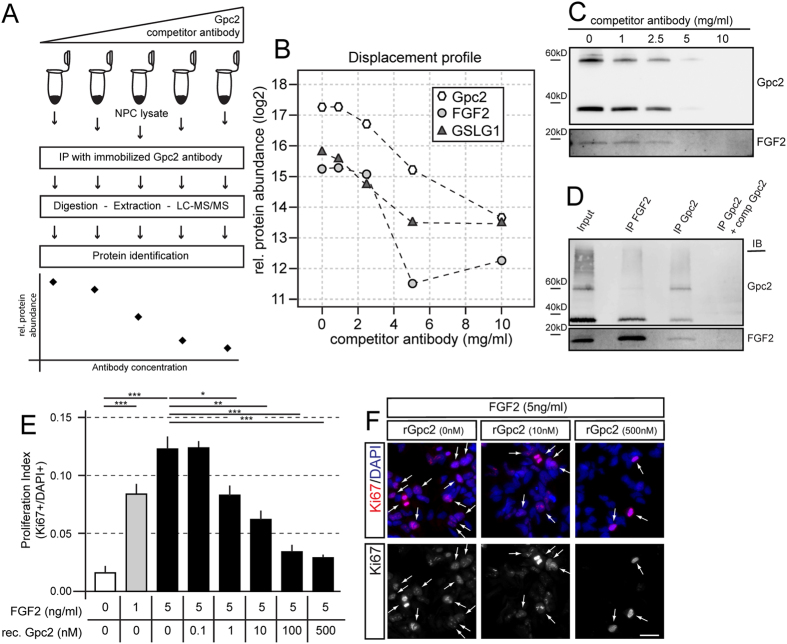
Glypican-2 binds FGF2 and modulates FGF2-mediated progenitor cell proliferation. (**A**) Experimental work flow for immunocompetitive capture mass spectrometry (ICC-MS). (**B)** ICC-MS displacement profile of neural progenitor cells cultured for 21 days under differentiation conditions. FGF2 and GSLG1 show a statistically significant concentration-dependent decrease in signal. (**C)** Eluted fractions of the Gpc2 immunocompetitive precipitations. Cell lysates were pre-incubated with 0, 1, 2.5, 5 and 10 μg/ml of free Gpc2 antibody before immunoprecipitation with the Gpc2-specific antibody (clone 2/43). The detection of the FGF2 protein decreased as the concentration of free competitor antibody increased, similar to Gpc2, thereby confirming the specificity of the FGF2 co-immunoprecipitation. (**D)** FGF2 is detected in the bound fraction of the FGF2 immunoprecipitation. Gpc2 is co-captured with FGF2. An IP of a lysate pre-incubated with 10 μg/ml of free Gpc2 antibody is also shown as a control. Input: cell lysate. (**E)** Increasing amounts of FGF2 promote progenitor cell proliferation. Recombinant Gpc2 counteracts the proliferative effect of FGF2. (**F)** Representative images of proliferating neural precursor cells (arrows) cultured in the presence of increasing amounts of Gpc2. Error bars represent the SEM. Scale bars = 10 μm. p < 0.05 = *; p < 0.01 = **; p < 0.001 = ***.

We performed immunocompetitive capture mass spectrometry (ICC-MS) to identify the proteins that bind Gpc2 and may explain the link between neurogenesis and Gpc2 (Fig. 2a)^20^. Competition between free and capture antibodies leads to increased specificity compared to classical IP with a control^20^. This technique results in a concentration-dependent displacement profile that is shared by the target protein and its interacting partners (Fig. 2a). We applied a competitor antibody against Gpc2 to differentiating NPC lysates to obtain a specific displacement profile for Gpc2 (Fig. 2b). MS-based quantification followed by a robust statistical analysis of the co-immunoprecipitated proteins led to the identification of several proteins that showed a significant displacement in addition to Gpc2 (Supp. Table 1a). FGF2 and GSLG1, a ligand and a receptor in the FGF signaling cascade, respectively, were pulled down with Gpc2 and showed a specific displacement profile (Fig. 2b,c). We performed a reverse pull-down using FGF2 as a bait to further validate that FGF2 bound to Gpc2 and confirmed that Gpc2 was co-immunoprecipitated with an FGF2-specific antibody, supporting the hypothesis that both proteins specifically interact with each other (Fig. 2d).

Fibroblast Growth Factor has been shown to play an important role in the control of adult NPC proliferation^21^. We co-cultured hNPCs in the presence of FGF2 in combination with increasing amounts of recombinant Gpc2 to determine whether Gpc2 had an effect on FGF-mediated proliferation. FGF2 leads to a net increase in progenitor cell proliferation, as determined by the number of Ki67 ^+^ cells within the total population (Fig. 2e,f). This pro-proliferative effect was inhibited by the addition of soluble Gpc2 in a concentration-dependent manner (Fig. 2e,f). Thus, soluble Gpc2 inhibits FGF2-mediated proliferation, potentially by sequestering FGF2 and subsequently reducing its availability.

### Glypican-2 expression is confined to neurogenic regions in the adult brain, correlates with the postnatal age-induced reduction in neurogenesis and is detected in rodent cerebrospinal fluid

**Figure 3:**
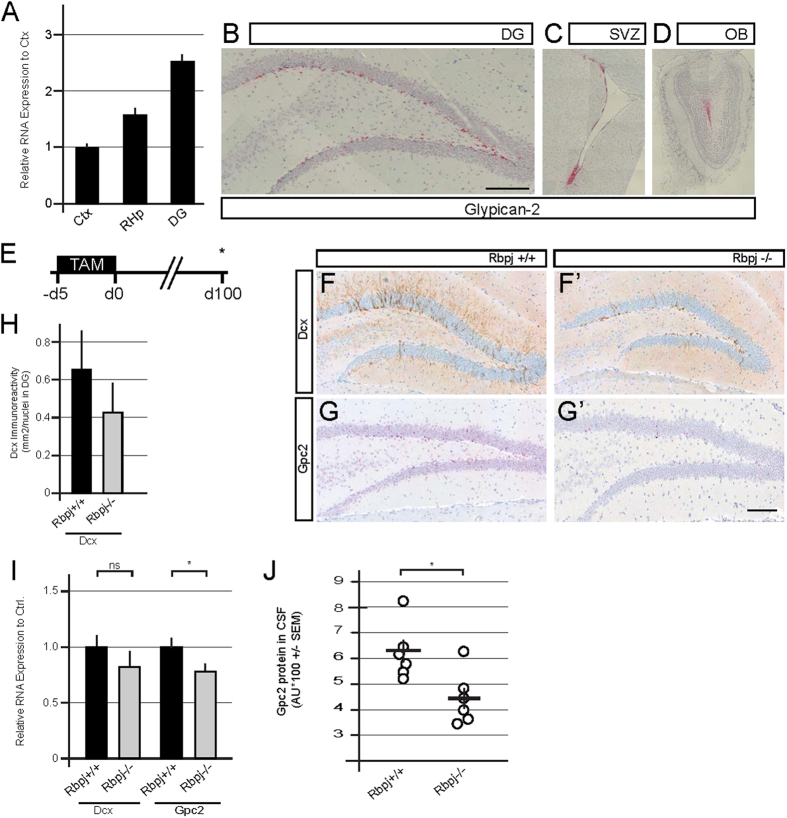
Glypican-2 expression is restricted to the neurogenic regions in the adult rodent brain. Genetic ablation of neurogenesis results in a strong reduction of Glypican-2 expression. (**A)** Expression of Gpc2 mRNA in the micro-dissected dentate gyrus (DG) and residual hippocampus (RHp) was compared to expression in the cortex (Ctx) of adult mice. Gpc2 is expressed at the highest levels in regions with continuous adult neurogenesis.(**B–D)** *In situ* hybridization of Gpc2 RNA in the adult mouse brain (w7). Gpc2 expression is restricted to the subgranular zone of the DG **(B)**, the lateral ventricles of the SVZ **(C)** and the rostral migratory stream entering the OB **(D)**. (**E)** Experimental design for inducing a loss of neurogenesis by Hes5::CreERT^2^-mediated ablation of Rbpj^fl/fl^. (**F–F’)** Representative Dcx immunostaining in Rbpj^+/+^ Ctrl animals **(F)** and Rbpj^−/−^ mutants **(F’). (G–G’)** Gpc2 *in situ* hybridization in Rbpj^+/+^ Ctrl animals **(G)** and Rbpj^−/−^ mutants **(G’). (H)** Quantification of Dcx immunoreactivity. The ablation of Rbpj results in a reduction in Dcx immunoreactivity *in vivo*. (**I)** Relative expression of Dcx and Gpc2 mRNAs in micro-dissected hippocampi from the Rbpj^−/−^ mutants. (**J)** CSF levels of Gpc2 protein. The ablation of Rbpj results in a significant decrease in the levels of Gpc2 in mouse CSF. Ctx, cortex; RHp, residual hippocampus; DG, dentate gyrus; SVZ, subventricular zone; OB, olfactory bulb. The error bars shown in J represent the SEM. The error bars shown in A, H and I represent CI. Scale bar = 200 μm. p < 0.05 = *; p < 0.01 = **; p < 0.001 = ***.

In adult mammals, NSCs and their progeny are located within two discrete regions of the brain, the subgranular zone (SGZ) of the dentate gyrus (DG) and the subventricular zone-rostral migratory stream-olfactory bulb region^22^. According to the Allen Brain Atlas, Gpc2 expression substantially enriched in these two regions (http://mouse.brain-map.org)^16^. We performed *in situ* hybridization (ISH) in neurogenic regions from adult mouse brains to corroborate these findings. The Gpc2 mRNA was predominantly located within the subgranular zone (SGZ) (Fig. 3b), the dorsal and ventral regions of the subventricular zone (SVZ) (Fig. 3c) and within the rostral migratory stream (RMS) (Fig. 3d), indicating that expression is restricted to cells located in the adult neurogenic brain regions. The ISH data were confirmed by RT-PCR using micro-dissected mouse brains to compare RNA expression between the DG, residual hippocampus and the cortex (Fig. 3a). We observed the strongest expression in the DG compared to the residual hippocampus and the non-neurogenic regions of the cortex (Fig. 3a).

Hippocampal neurogenesis dramatically decreases during the initial postnatal period after birth^23^,^24^. We micro-dissected the hippocampi from rats at early postnatal developmental stages to determine whether a decrease in postnatal neurogenesis is reflected by a similar decrease in Gpc2 expression. Neurogenesis, as determined by Dcx expression, sharply decreased during postnatal development. Gpc2 expression exhibited the same decrease in expression and showed the strongest reduction after postnatal day 10 (Supp. Fig. 3a).

Proteins within cerebrospinal fluid are used as biomarkers for disease diagnosis and progression^25^,^26^,^27^,^28^,^29^, and we recently showed that the levels of the Dcx protein in the CSF can be utilized as a proxy marker of neurogenesis^30^. However, Dcx expression was not restricted to the neurogenic regions and was not detectable after postnatal day 40^30^. As Gpc2 is expressed in a more restricted pattern than Dcx in rodents and is proteolytically cleaved and secreted (Fig. 1e,f), we reasoned that it may have superior physical properties as a CSF biomarker compared with Dcx. Thus, we measured the levels of Gpc2 protein in rodent CSF to determine whether the abundance of this protein reflected the degree of adult neurogenesis. We observed a gradual decrease in the CSF levels of Gpc2 protein in an age-dependent manner, from postnatal day 5 (P5) to postnatal day 40 (P40) with sufficient levels Gpc2 present in CSF for quantification (Supp. Fig. 3b).

We conclude that the CSF levels of Gpc2 protein during postnatal periods reflect the gradual decrease in neurogenesis, and we next tested whether Gpc2 has the potential as a CSF biomarker for adult neurogenesis.

### Genetic ablation of neurogenesis correlates with Gpc2 levels in the CSF

We analyzed the effect of genetically mediated loss of neurogenesis on the CSF levels Gpc2 to identify a direct connection between adult neurogenesis and CSF levels of Gpc2 protein. Ablation of the Notch signaling mediator Rbpj in adult neural stem cells of the DG leads to the long-term loss of hippocampal neurogenesis^31^,^32^. We genetically deleted Rbpj in Hes5^+^ NSCs and also observed a significant reduction in the number of Dcx-positive cells in the DG (Fig. 3f-g’)^33^. Genetic depletion of Rbpj also led to reduced Dcx and Gpc2 expression as shown by qPCR using micro-dissected hippocampi (Fig. 3i). We next analyzed the CSF levels of Gpc2 protein to determine whether the loss of neurogenesis can be monitored. We observed a significant reduction in the CSF levels of Gpc2 in the Rbpj^−/−^ group compared to the control animals (Fig. 3j). To corroborate our findings, we used another genetic model to arrest adult neurogenesis and exclude the possibility that Notch signaling directly regulates Gpc2 independent of its effect on adult neurogenesis (Supp. Fig. 2). We assessed whether the arrest of adult hippocampal neurogenesis with a genetic model of ablation (GFAP-TK model mice) resulted in a decrease in the levels of Gpc2 in the CSF and DG. Therefore, a 4-week GCV treatment was applied to GFAP-TK mice, resulting in targeted ablation of the stem cell population in the DG with a concomitant loss of neurogenesis^34^. A strong reduction in the number of Dcx^+^ cells was observed in the DG, reflecting the loss of neurogenesis (Supp. Fig. 2b). The effect on neurogenesis was confirmed by the reduction in Dcx mRNA expression in micro-dissected hippocampi (Supp. Fig. 2c). Concomitantly, the expression of Gpc2 mRNA was significantly reduced in micro-dissected hippocampi upon ablation of neurogenesis (Supp. Fig. 2d). The reduction in active neurogenesis was reflected in a significant reduction in the CSF levels of Gpc2 protein (Supp. Fig. 2d). Based on the data obtained from the genetic ablation experiments, Gpc2 is linked to adult neurogenesis, and alterations in neurogenesis can be monitored by measuring CSF levels of Gpc2 protein.

### Physiological regulation of neurogenesis results in a concomitant modulation of Glypican-2 CSF levels that correlate with the number of immature neurons

Adult hippocampal neurogenesis is dynamically regulated by physiological stimuli, including physical exercise, antidepressant treatment, age and pathological conditions, such as stress^35^,^36^,^37^. We tested several paradigms that modulate adult hippocampal neurogenesis to analyze whether the changes in neurogenesis are reflected in the CSF levels of Gpc2.

**Figure 4:**
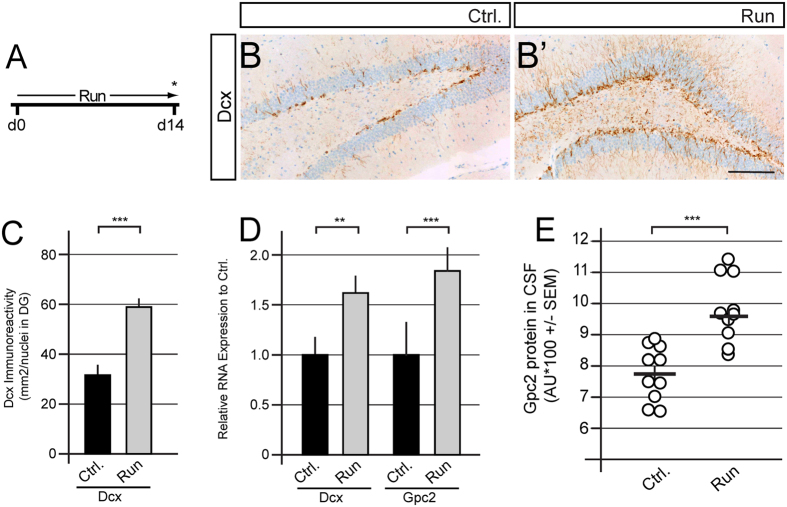
Physical exercise-induced neurogenesis can be monitored by examining the increase in Gpc2 CSF levels. (**A**) Experimental paradigm for the running-mediated induction of neurogenesis; free access to a running wheel was provided for a period of 14 days. (**B-B’)** Representative images of Dcx immunostaining. (**C**) Quantification of Dcx immunoreactivity and Gpc2 *in situ* labeling after running-induced neurogenesis. The expression of both Dcx and Gpc2 is induced by voluntary physical exercise. (**D**) Expression of Dcx and Gpc2 mRNAs in micro-dissected hippocampi. (**E**) CSF levels of Gpc2 after voluntary exercise. Running increases the CSF levels of Gpc2, which correlate with the extent of neurogenesis. Error bars shown in E represent the SEM. Error bars shown in C and D represent CI. Scale bar = 100 μm. p < 0.05 = *; p < 0.01 = **; p < 0.001 = ***.

When provided free access to a running wheel, the running group showed a significant increase in the number of immature neurons, as measured by Dcx immunoreactivity (Fig. 4a,b,b’). Furthermore, running-induced neurogenesis correlated with increased expression of Dcx and Gpc2 mRNAs in the micro-dissected DG, reflecting the increased number of Dcx^+^ cells (Fig. 4d). In addition, running induced a significant increase in the CSF levels of Gpc2 protein compared to sedentary animals (Fig. 4e). Thus, the CSF levels of Gpc2 reflect the extent to which physical exercise induces adult neurogenesis.

### Pharmacological induction of neurogenesis can be monitored using the CSF levels of Glypican-2

**Figure 5:**
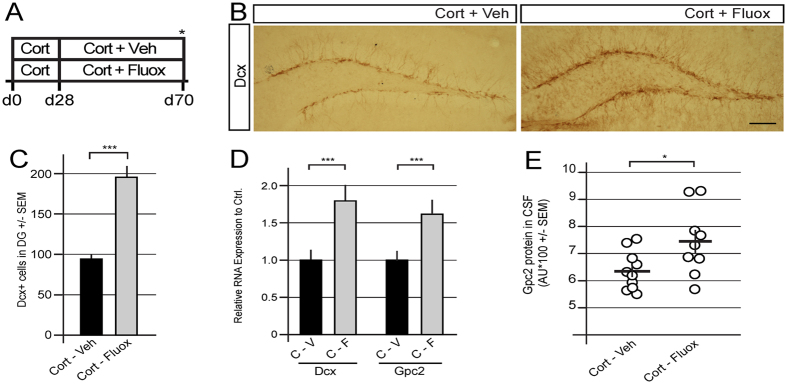
Pharmacological induction of neurogenesis can be monitored using the CSF levels of Glypican-2. (**A)** Experimental paradigm for fluoxetine-mediated induction of neurogenesis. (**B)** Representative images of Dcx expression. (**C)** Quantification of the number of Dcx-positive cells in the DG. Fluoxetine treatment leads to increased numbers of Dcx^+^ cells in the DG. (**D)** Expression of Dcx and Gpc2 mRNAs in micro-dissected dentate gyri. Fluoxetine treatment leads to increased expression of Dcx and Gpc2 mRNAs. (**E)** CSF levels of Gpc2 after fluoxetine-mediated induction of neurogenesis. Fluoxetine treatment leads to increased Gpc2 CSF levels. Error bars shown in C and E represent the SEM. Error bars shown in D represent the CI. Scale bar = 100 μm. p < 0.05 = *; p < 0.01 = **; p < 0.001 = ***.

Increased hippocampal neurogenesis after chronic monoaminergic antidepressant (AD) treatment is required for behavioral activity^37^,^38^,^39^,^40^,^41^. Gpc2 expression was monitored in anxious/depressed mice (CORT model) that had been treated with fluoxetine for one month to determine whether the CSF levels of Gpc2 can be used to monitor the pharmacological induction of neurogenesis after a chronic antidepressant treatment (Fig. 5a). The fluoxetine-induced increase in adult hippocampal neurogenesis was confirmed by the increase in the number of Dcx^+^ cells in the adult DG (Fig. 5b,c) as well as changes in the expression of Dcx and Gpc2 mRNAs in the micro-dissected DG (Fig. 5d). This increase was paralleled by an increase in the CSF levels of Gpc2 protein in response to the antidepressant treatment (Fig. 5e). Thus, CSF levels of Gpc2 may be used to monitor the pharmacological induction of neurogenesis and potentially reflect the stimulation of adult hippocampal neurogenesis.

### The age-mediated reduction in adult neurogenesis is reflected in the CSF levels of Glypican-2 in rodents

**Figure 6:**
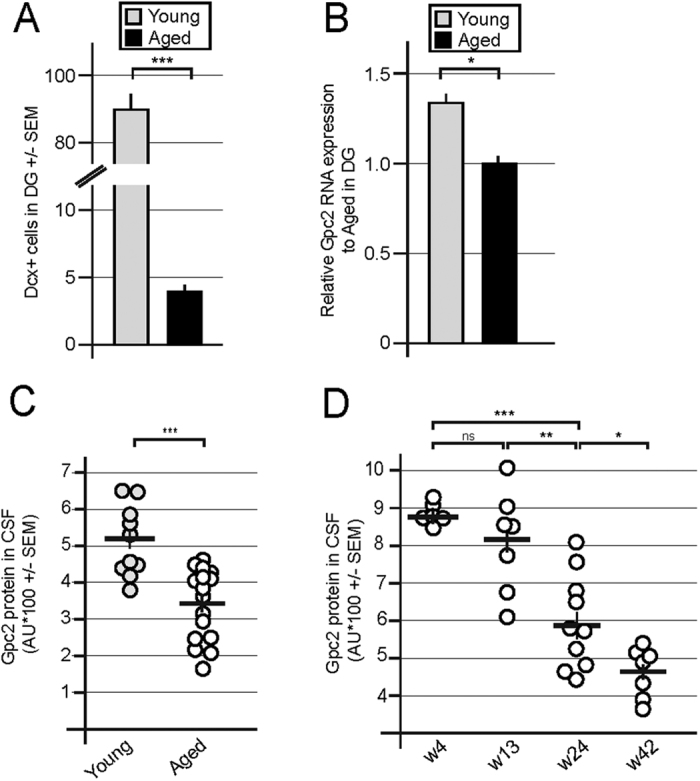
The age-mediated reduction in adult neurogenesis is reflected in the CSF levels of Glypican-2 in rodents. (**A)** Quantification of the number of Dcx-positive cells in the DG in young and aged animals. Aging leads to a marked reduction in the number of Dcx^+^ cells in the DG. (**B)** Relative expression of Gpc2 mRNA in micro-dissected dentate gyri from young animals compared to aged animals. (**C)** Gpc2 levels in the CSF from young mice compared to aged animals. (**D**) Gpc2 levels in the CSF from rats decrease during the early postnatal period and are still detectable in postnatal week 42. Error bars shown in C and D represent the SEM. Error bars shown in A and B represent CI. p < 0.05 = *; p < 0.01 = **; p < 0.001 = ***.

Aging leads to a reduction in adult neurogenesis, as determined by loss of proliferating neural precursor cells and the reduction in total Dcx^+^ progenitors^23^,^31^,^42^,^43^. We compared young adult mice (approximately 10 weeks of age) to aged mice (>52 weeks of age) and observed a substantial reduction in the number of Dcx-positive cells within the DG (Fig. 6a). This age-dependent decrease was reflected by a significant reduction in Gpc2 expression in the micro-dissected DG from aged mice (Fig. 6b). Furthermore, the age-dependent reduction of neurogenesis was monitored using the CSF levels of Gpc2 protein (Fig. 6c). Age-dependent reduction and preserved expression of Gpc2 in into adulthood was also observed in rat CSF by analyzing Gpc2 protein abundance at defined postnatal periods (Fig. 6d).

Based on these results, the CSF levels of Gpc2 protein can be used as a CSF biomarker to detect changes in the extent of adult neurogenesis modulated by physical exercise, antidepressant treatment and aging.

### Glypican-2 is detectable in human cerebrospinal fluid

**Figure 7:**
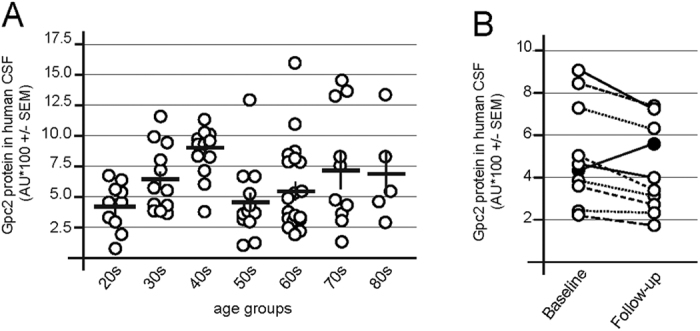
Gpc2 is present in human CSF and shows a moderate decrease in a aging longitudinal cohort. (**A)** Gpc2 protein was detected in human CSF samples grouped by age. Each dot represents an individual human donor. (**B)** Longitudinal analysis of the CSF levels of Gpc2 in individual samples. Baseline measurements were compared to a 3-year follow-up measurement. Error bars shown in C and D represent the SEM. Error bars shown in A and B represent CI. p < 0.05 = *; p < 0.01 = **; p < 0.001 = ***.

As an initial test to assess the feasibility of measuring Gpc2 protein levels in human CSF, we measured the levels of Gpc2 protein in commercially available CSF samples obtained from 83 individuals (Fig. 7a). Gpc2 was detected in all human CSF samples analyzed, although the expression levels were highly variable and lacked a correlation with age when the samples were grouped accordingly (Fig. 7a).

Inter-subject variability might prohibit a correlation with age and potential technical sources of variability cannot be excluded in commercially available CSF samples. We therefore analyzed CSF Gpc2 levels in a small subset of human CSF samples for which longitudinal CSF sampling has been performed in order to reduce potential technical sources of variability and to better address intra-subject variability. When Gpc2 levels in three-year follow-up samples were compared to the baseline samples, we observed low intra-subject variability and a decrease in the CSF levels of Gpc2 in 9 out of 10 individuals (Fig. 7b).

Our initial findings in human CSF support to the prospective use of Gpc2 as a potential CSF biomarker for human adult neurogenesis.

## Discussion

Current standard methods for monitoring adult neurogenesis are based on a retrospective analysis of *post mortem* tissue^44^,^45^. The first report on human adult neurogenesis used brain samples from patients with cancer who had received a single injection of BrdU for diagnostic purposes^3^. Recently, a combination of fluorescent activated cell sorting of NeuN-positive neurons with ^14^C-dating to date the nuclear content of these neurons has been used to show that neurogenesis exists in several regions of the adult human brain^1^,^46^,^47^.

Currently, efforts to identify biomarkers that reflect the generation of newborn neurons in living humans have been hampered by the correlative nature, sensitivity or reproducibility of the results^7^,^30^,^48^,^49^. The lack of a direct marker for adult neurogenesis has precluded studies of the functional relevance of adult neurogenesis in human health and disease.

The detection of proteins in CSF shows promise as a reflection of the physiological and pathophysiological changes in the central nervous system. The beta-amyloid and Tau proteins have proven to be valuable diagnostic biomarkers for Alzheimer’s disease^50^,^51^. As shown in our recent study, CSF levels of the doublecortin protein (Dcx) can be used to measure neurogenesis^30^. However, the Dcx levels decrease below the limit of detection early during the life of an organism, limiting the utility of this method. Here, we identified Gpc2 as a CNS-specific protein that may enable analysis of the extent of neurogenesis by measuring its levels in CSF, taking advantage of its intrinsic properties, namely transient expression in progenitor cells, cleavage of the protein and concomitant release into the extracellular milieu. The CSF levels of Gpc2 protein reflect the induction of neurogenesis after pharmacological treatment and physical exercise (Figs 4 and 5). As physical exercise induces systemic effects on an organism, such as the induction of hippocampal blood flow and release of neurotrophins and other growth factors in the brain, we cannot attribute the full extent of Gpc2 CSF levels to DG neurogenesis^48^,^52^,^53^. Moreover, after genetically mediated loss of neurogenesis, Gpc2 expression in the adult DG and the CSF levels of Gpc2 are reduced (Fig. 3 and Supp. Fig. 2), implying a correlation between the CSF levels of Gpc2 and adult neurogenesis.

In preliminary experiments, we show that Gpc2 is present in human CSF but its levels are highly variable (Fig. 7). Little is known about the inter-individual variation of human adult neurogenesis but based on data obtained from rodent studies it is likely that lifestyle (exercise, illness, diet, etc.) and genetics can contribute dramatically to the rates of neurogenesis and affect individual CSF Gpc2 levels (for review, see ref. 54). Furthermore, profound variability can be due to technical aspects (eg. different CSF collection and processing procedures, repeated freeze-thaw cycles, prolonged or inaccurate storage of samples) and has been shown for other CSF analytes (for review, see ref. 55).

To this end, we tested Gpc2 levels in a small cohort of non-demented aged individuals (Fig. 7b). CSF samples have been collected in controlled setting which reduces the variability due to technical aspects. Analysis of longitudinal human CSF samples (baseline and after a 3 year follow-up) reduces the impact of inter-individual biological factors such as genetics and lifestyle differences. While the sample size in this initial experiment was limited, we observe low variability of CSF Gpc2 level within subjects. The modest reduction observed in 9 of 10 individuals seems plausible and could represent potential changes within the CNS that correlate with adult neurogenesis such as age-dependent changes in cognition observed in elderly populations^56^,^57^.

Our data highlights the novelty and the advantage of Gpc2 compared to DCX as a CSF biomarker of the process of adult neurogenesis. Unlike DCX, Gpc2 expression is not restricted to early postnatal stages of development, which enabled us to directly assess the effect of altered adult neurogenesis on Gpc2 CSF levels in animal models (Fig. 3e–J). Gpc2 levels are detectable in rodents CSF up to 2 years of age and in CSF samples derived from human donors up to 90 years of age (Figs 6 and 7). Our conclusion from these studies is that Gpc2 immunoassay enables precise and quantitative measurements of adult neurogenesis across ages from rodents to humans.

Our first experiments using human CSF samples indicate that while CSF Gpc2 levels show high variability between subjects, the intra-subject variability is low. Further longitudinal experiments with samples from large, well-characterized cohorts of individuals with and without central nervous system diseases or under controlled conditions that modulate neurogenesis are required to validate this initial finding and to assess utility of CSF Gpc2 analysis in the clinic.

Notably, another member of the Glypican family, Gpc3, may serve as a diagnostic serum marker of hepatocellular carcinomas^58^, indicating that HSPGs can be used as biomarkers.

From a biological perspective, we provide insights as to how a specific HSPG may control the availability of growth factors in the neurogenic niche. Cellular and molecular components provide a specific local microenvironment in the adult SGZ that provides the necessary signals to maintain the stem cell population and to support neural differentiation and maturation. Microglia, astrocytes, neurons and the local vasculature provide direct cell-to-cell contacts, neurotrophic support, neurotransmitters and growth factors to control individual steps in the process of neurogenesis^59^. Environmental changes that modulate neurogenesis, such as physical exercise, likely function by inducing changes in specific trophic factors within the niche^53^. In addition, exercise leads to changes in the local brain vasculature and permeability of the blood brain barrier, resulting in higher concentrations of circulating growth factors^60^,^61^,^62^.

HSPGs constitute part of the local environment in the adult DG and have also been shown to play a critical role in the modulation of key signaling pathways for secreted factors, such as Wnt, Shh, BMP and FGF during mammalian development^63^. The exogenous application of Gpc2 has recently been shown to promote the differentiation of mouse dorsal root ganglion neurons^64^. As shown in our study, Gpc2 mediates its effects on neural differentiation by sequestering FGF2 (Fig. 2). Interactions between Gpc2 and FGF2 have been reported in neural cells of the developing rat brain, indicating that a similar process may occur during neural differentiation *in vivo*^65^.

We hypothesize that Gpc2 functions as a niche factor that regulates the availability of FGF2 and thus modulates progenitor cell proliferation. The binding of Gpc2 to FGF2 may reduce the local concentrations within the niche, potentially resulting in a negative feedback loop that reduces the net amount of proliferation. As shown in our study, the presence of high CSF levels of Gpc2 and abundant expression of membrane-bound HSPGs in the brain^66^ support the hypothesis that strategies controlling the local amount of mitogens influence neurogenesis^67^. In conclusion, Gpc2 may represent an important component of the neural stem cell niche. We expect that Gpc2 protein expression is restricted to cells during the process of neurogenesis based on, (a) the restricted expression to adult neurogenic niche shown by *in-situ*-hybridization, (b) its correlation with expression changes in DCX as a marker of neural blast and immature neurons, and (c) its reduced CSF protein levels after ablation of neurogenesis in different transgenic models. Due to technical limitations, it was not possible to perform a stage specific cellular analysis of Gpc2 protein expression. Since a previous study reported that Gpc2 is a Sox11 target gene, it is likely to be restricted to Sox11^+^ neuronal precursors and immature neurons, but this will require further confirmation^68^.

In summary, we have identified a CNS-specific protein, Glypican-2, that may modulate proliferation by sequestering FGF2 in the neurogenic niche and may be used as a CSF biomarker for adult neurogenesis. Further studies focusing on characterization of the CSF Gpc2 signal and stability, as well as analyses in diseases in which neurogenesis is affected, are required to validate Gpc2 as a biomarker for neurogenesis. We are tempted to speculate that changes in CSF Gpc2 levels may be observed in depression and Alzheimer’s disease, human diseases in which a reduction in adult neurogenesis has been described^69^,^70^. A CSF biomarker reflecting the extent of neurogenesis in the brain is highly desirable and would be a valuable tool for monitoring treatment efficacy in patients with brain diseases involving neurogenesis. Moreover, the CSF biomarker would open new possibilities for investigating the response of the brain to trauma and insults and would be useful in the development of new therapies for degenerative brain diseases.

## Materials and Methods

### Human neural progenitor cultures

The conditions used for the neural progenitor cultures have been previously described^71^. Briefly, cells were maintained in dishes coated with poly-L-ornithine (Sigma) and laminin (Roche). Neural progenitor cells were cultured in basic medium composed of equal volumes of DMEM:F12 Glutamax medium and Neurobasal medium (Gibco, Invitrogen) supplemented with 2% B27 (Gibco, Invitrogen), 1% N2 (Gibco, Invitrogen), 0.1% beta-mercaptoethanol (Gibco, Invitrogen), 10 ng/ml FGF (Peprotech), 10 ng/ml EGF (R&D Systems) and 20 ng/ml BDNF (Peprotech). Cells were replated on coated dishes at a density of 45,000 cells/cm^2^ and maintained in basic medium supplemented with 20 ng/ml BDNF (Peprotech) to induce differentiation. Alternatively, differentiation was induced by replating NPCs at a density of 12,000 cells/cm^2^ and culturing in basal medium supplemented with 200 ng/ml Shh (Peprotech), 100 ng/ml FGF8 (Peprotech) and 100 μM ascorbic acid (Sigma) for 7 days, with subsequent replating in basal medium supplemented with 20 ng/ml BDNF (Peprotech), 10 ng/ml GDNF (Peprotech), 0.5 mM cAMP (BIOLOG Life Science) and 100 μM ascorbic acid (Sigma) at a density of 45,000 cells/cm^2^.

Neural progenitor cells were cultured in basic medium composed of equal volumes of DMEM:F12 Glutamax medium and Neurobasal medium (Gibco, Invitrogen) supplemented with 2% B27 (Gibco, Invitrogen); 1% N2 (Gibco, Invitrogen); 0.1% beta-mercaptoethanol (Gibco, Invitrogen); 0, 1 or 5 ng/ml FGF2 (Peprotech); and 0, 0.1, 1, 10, 100 or 500 nM recombinant human Gpc2 (R&D Systems) to analyze the effect of Gpc2 on FGF2-mediated proliferation.

### Mass spectrometry analysis

#### Discovery mass spectrometry analysis

In the initial discovery proteomics step, biological triplicates of NSCs (approximately 15 × 10^6^ cells per condition) were harvested after t = 0, 1, 2, 4, 7, 14, 21 or 28 days. After lysis by sonication, a crude membrane fraction was obtained by centrifugation, washed in high salt/high pH to deplete the soluble proteins and further fractionated by SDS-PAGE. Each lane was divided into 12 bands that were subjected to in-gel trypsinization and individually analyzed on a nanoLC-MS/MS using a Thermo LTQ-Orbitrap mass spectrometer (Thermo Fisher Scientific).

#### Glypican-2 interaction profiling by immunocompetitive capture

In-house-generated anti-Glpc2 antibodies were used for Western blot detection, IP and the competition experiments (clone 2/43). Anti-FGF2 from Abcam (ab126861) was used for both IP and blot detection. The Gpc2 immunocompetitive capture experiment was performed as previously described^20^. Briefly, the anti-Gpc2 resin was incubated with 21-day differentiated NPC lysates (850 μg total protein per condition) for 1 h. For the competition experiments, the lysates were pre-incubated with increasing amounts of free anti-Gpc2 antibody (0, 100, 250, 500 and 1000 ng) in triplicate for 1 h. The eluates were separated on SDS-PAGE gels, and four bands spanning 20 to 120 kDa were subjected to in-gel trypsinization. Samples were analyzed with a Nanoflow Easy-nLC system (Proxeon) connected to an LTQ-Orbitrap Velos (Thermo Fisher Scientific).

### Animals

All experiments involving mice and rats were performed by authorized investigators following national and European ethical guidelines. Young adult mice (C57BL/6 N from Charles River Laboratories, France) between 8 and 10 weeks of age were used for the experiments, unless stated otherwise. Mice were maintained on a 12-h day/night cycle with adequate food and water, according to the guidelines from local veterinary authorities under license numbers 2012–0016/2584 (Ethical Commission Kanton Basel, Switzerland). Experiments using the Hes5::CreER^T2^ RBPjk^fl/fl^ model were conducted under SPF conditions according to institutional regulations under license number 2537 (Ethical Commission Kanton Basel, Switzerland). All testing of the corticosterone model was conducted in compliance with the laboratory animal care guidelines and with protocols approved by the Institutional Animal Care and Use Committee (European Directive, 2010/63/EU for the protection of laboratory animals, permissions # 92-256B, authorization ethical committee CEEA n126 2012_098).

Rat tissues and cerebrospinal fluid (Sprague-Dawley) obtained at different postnatal developmental stages were purchased from QPS Austria GmbH.

### GFAP-TK mouse model

An agreement (license L-O 15-2015/0) between the NIH and the Université Paris-Sud provides UMRS 1178 with the use of transgenic mice that express herpes simplex virus thymidine kinase (TK) under control of the glial fibrillary acidic protein (GFAP) promoter (GFAP-TK mice), as previously described^34^ and developed in the laboratory of Dr. Heather Cameron of the National Institute of Mental Health (NIMH).

### Ganciclovir administration

Ganciclovir (Rodent Diet, Grain-Based, Valganciclovir, VGCV, 165 mg/kg; Custom Animal Diets, LLC) was administered to the treatment group in the chow for a period of 4 weeks using the method reported in previous studies^34^,^72^. GFAP-TK-positive mice and control littermates received Valganciclovir. VGCV (Roche, Indianapolis, IN), the L-valyl ester of ganciclovir, has a high (approximately 85%) bioavailability and is rapidly converted into ganciclovir by intestinal and hepatic esterases after oral administration. After phosphorylation by HSV-TK, ganciclovir is toxic to proliferating cells in S phase of mitosis.

### Corticosterone model

The hypothalamic-pituitary-adrenal (HPA) axis is often dysregulated in clinical depression. The glucocorticoid levels are exogenously increased in this model. This chronic elevation of corticosterone (CORT) levels results in dysregulation of the HPA axis. For example, a blunting of the HPA axis in response to stress is observed in CORT-treated mice, as shown by the marked attenuation of stress-induced CORT levels^38^,^39^. This model reliably induces anxious and depressive-like behaviors in mice. The dose and duration of the CORT treatment were selected based on previous studies^38^,^39^. CORT (35 μg/ml, equivalent to approximately 5 mg/kg/d) or vehicle (0.45% β-CD) was provided in the drinking water protected from light in opaque bottles *ad libitum*. CORT-treated water was changed every 3 days to prevent possible degradation. Fluoxetine hydrochloride (160 mg/ml, equivalent to 18 mg/kg/day) was purchased from Anawa Trading (Zurich, Switzerland) and dissolved in a 0.45% β-CD/corticosterone solution. Thereafter, during the administration of β-CD or corticosterone, mice were treated with vehicle (0.45% β-CD) or fluoxetine in the drinking water, as previously described^38^.

### Hes5::CreER^T2^, RBPjk^fl/fl^ mouse model

*Hes5::CreER*^*T2*^ and floxed *RBPjk* mice have been previously described^33^,^73^.

### Tamoxifen administration

Stock solutions of tamoxifen (Sigma) were prepared at a concentration of 20 mg/ml in corn oil (Sigma). Adult mice were i.p. injected with 2 mg/day tamoxifen once per day for 5 consecutive days to induce recombination and were euthanized for analysis 21 or 100 days after the last injection. The brains were prepared for further processing as described below.

### Running

The running experiment has been previously described^35^,^36^. Briefly, in two sets of experiments, 2-month-old female mice were randomly divided into 2 groups. The control group was placed in standard housing, and the running group was housed with free access to a running wheel for a period of 14 days. In both experimental groups, the mice were housed in pairs in each cage. At the end of the experiment, the animals were deeply anesthetized by an i.p. injection of pentobarbital solution (150 mg/kg BW), cerebrospinal fluid (CSF) was collected and brains were processed as described below.

### Collection of cerebrospinal fluid and Glypican-2 immunoassay

For CSF collection, the mice were deeply anesthetized by an intraperitoneal (i.p.) injection of pentobarbital solution (150 mg/kg body weight). CSF was isolated by puncturing the cisterna magna with a narrow microcapillary needle (Hirschmann). The skin and musculature overlaying the cisterna magna were carefully removed to expose the arachnoid membrane covering the cistern. The surrounding area was gently cleaned to remove the residual blood and interstitial fluid. Upon puncture of the membrane, CSF was readily collected by capillary forces. In cases with strong blood contamination, the sample was excluded from further analysis. CSF was snap-frozen on dry ice until further analysis. Immunoassays for Gpc2 were performed using the AlphaLISA bead-based technology (Perkin Elmer) according to the manufacturer’s protocol. Briefly, AlphaLISA acceptor beads were conjugated with a novel monoclonal antibody against mouse Gpc2, mAb4/7, to obtain a final concentration of 5 mg/ml of conjugated antibody in PBS with 0.05% Proclin300 (Sigma). Biotinylated mAb4/15, a second novel monoclonal antibody against mouse Gpc2, was used to detect Gpc2. The rodent CSF samples were diluted in AlphaLISA HiBlock buffer (Perkin Elmer) in the presence of 1% normal human serum (Sigma) to analyze Gpc2 levels. Acceptor-bead coupled mAb4/7 (10 μg/ml) and 1 nM biotinylated mAb4/15 were incubated with the corresponding sample for 3 h at RT. Streptavidin-conjugated donor beads were added to a final concentration of 40 μg/ml and incubated for an additional 30 min at RT in the dark. The plates were analyzed in a 2104 EnVision Multilabel Reader (Perkin Elmer). Recombinant mouse Glypican-2 (R&D Systems) was used as a positive control. Selectivity for Glypican-2 was shown by the absence of immunoreactivity for recombinant Glypican-1, Glypican-3 and Glypican-4.

Biotinylated AF2304 (R&D Systems) was used at a final concentration of 0.2 nM in combination with a final concentration of 10 μg/ml novel mAb2/32 against human Gpc2 coupled to acceptor beads to detect human Glypican-2. The assay was performed in AlphaLISA HiBlock buffer without additional supplements to detect human Gpc2.

Human CSF samples were purchased from Bioreclamation IVT, NY, USA. Recombinant human Glypican-2 (R&D Systems) was used as a positive control. In addition, subjects of both sexes aged 40 years or above were recruited from two different clinical centers in France and Sweden after the protocol was approved by the ethics committee of the corresponding countries: CCPPRB Alsace I Strasbourg, University Clinic Huddinge and Uppsala. All procedures were conducted according to the principles expressed in the Declaration of Helsinki, and all subjects provided written informed consent. In cases where the patients were considered not to have the capacity to consent, informed consent was provided by their relatives.

### Tissue preparation, immunohistochemistry and *in situ* hybridization

After CSF collection, the brains were removed from the skull and split into the two hemispheres. One hemisphere was fixed by a 24-h immersion in 10% neutral-buffered formalin for immunohistochemistry. The other hemisphere was further micro-dissected to prepare the dentate gyrus or respective subanatomical brain regions. Tissues were snap-frozen on dry ice for subsequent RNA analyses. For histology and *in situ* hybridization, coronal sections of equivalent regions of the caudal and rostral hippocampus were embedded in paraffin. Blocks containing the caudal hippocampus were prepared, and four series of slides per block were produced with a gap of 10 slides. IHC was performed on consecutive slides with an anti-DCX (rabbit polyclonal, Abcam) primary antibody on Ventana Discovery^®^ XT autostainer, and ISH was performed with an anti-Gpc2 probe (VS-Mm-GpC2, RNAscope^®^ technology, ACD) on Ventana Discovery^®^ Ultra autostainer.

### RNA preparation and RT-PCR analysis

Total RNA was isolated from snap-frozen brain tissues using TRIzol reagent (Life Technologies) according to the manufacturer’s protocol, and the RNA concentration was quantified using spectrophotometry. Four nanograms of total RNA were used for RT-PCR in a 384-well plate on a Roche Light Cycler LC480. The following Taqman assays (ABI) were used: Mm00549653 (Gpc2, mouse); Mm00438401 (Dcx, mouse); Mm00727586 (Tubb3, mouse); Mm99999915 (Gapdh, mouse); Rn00593367 (Gpc2, rat); Rn00584505 (Dcx, rat); Rn99999916 (Gapdh, rat). Relative quantification was based on expression of the GAPDH gene.

### Image analysis

All slides were scanned with Aperio ScanScope^®^. Semi-automated image analysis of immunostaining was performed with Definiens Tissue Studio™ software based on manually annotated regions of interest comprising the dentate gyrus.

### Statistical analysis

The data processing and statistical analyses of the ICC-MS experiments have been thoroughly described elsewhere^74^. In summary, after quality control of the identified peptide peaks, the log2 scaled extracted ion counts (XIC) were normalized and summed to calculate the relative protein abundance. Linear models and non-monotonic contrasts were used to fit the displacement of the protein abundance matrix with the concentration of free anti-Gpc2 antibody. *P* values were obtained from moderated *t* tests and adjusted for multiple tests according to the methodology described by Augustin *et al*.^74^. Statistical comparisons were conducted with a two-tailed unpaired Student’s *t*-test. Differences were considered statistically significant at *p < 0.05, **p < 0.01 and ***p < 0.001. The data are presented as the mean ± SEM. RNA expression data are presented as the mean ± CI.

Immunofluorescent staining with DAPI and Ki67 was manually traced using a 20x objective and analyzed with ImageJ software. Cell densities were expressed as the number of DAPI^+^ cells divided by the number of Ki67^+^ cells (proliferation index). Statistical comparisons were conducted with a two-tailed unpaired Student’s *t*-test. Differences were considered statistically significant at *p < 0.05, **p < 0.01 and ***p < 0.001. The data are presented as the mean ± SEM. For the CORT and GFAP-TK experiments, the data were analyzed using GraphPad Prism v6.0 f. For the CORT experiments, one-way ANOVA was applied to the data as appropriate. Significant main effects and/or interactions were analyzed using Fisher’s protected least significant difference (PLSD) post hoc tests or unpaired *t*-tests.

## Additional Information

**How to cite this article:** Lugert, S. *et al*. Glypican-2 level in cerebrospinal fluid predicts the status of adult hippocampal neurogenesis. *Sci. Rep.* **7**, 46543; doi: 10.1038/srep46543 (2017).

**Publisher's note:** Springer Nature remains neutral with regard to jurisdictional claims in published maps and institutional affiliations.

## Supplementary Material

Supplementary Information
