# Supplementary material for: Glypican-2 levels in cerebrospinal fluid predict the status of adult hippocampal neurogenesis
Source: Sci Rep. 2017 Apr 25;7:46543. doi: 10.1038/srep46543 (PMC5404329; doi:10.1038/srep46543)
Supplement: Supplementary Information [file srep46543-s1.pdf]

Supplementary Information

Glypican-2 levels in cerebrospinal fluid predict the status of adult hippocampal neurogenesis

Authors: S. Lugert[1#], T. Kremer[1#*], R. Jagasia[1*], A. Herrmann[2$], S. Aigner[1+], C. Giachino[3], I. Mendez-David[4], A. M. Gardier[4], J. P. Carralot[5], H. Meistermann[2], A. Augustin[2], M. D. Saxe[1§], J. Lamerz[2], G. Duran-Pacheco[2], A. Ducret[2], V. Taylor[3], D. J. David[4], and C. Czech[1]

[1] Roche Pharmaceutical Research and Early Development, NORD Discovery & Translational Area, Roche Innovation Center Basel, F. Hoffmann-La Roche Ltd, Grenzacherstrasse 124, 4070 Basel, Switzerland

[2.] Roche Pharmaceutical Research and Early Development, Pharmaceutical Sciences, Roche Innovation Center Basel, F. Hoffmann-La Roche Ltd, Grenzacherstrasse 124, 4070 Basel, Switzerland

[3.] Embryology and Stem Cell Biology, Department of Biomedicine, University of Basel, Mattenstrasse 28, CH-4058 Basel, Switzerland

[4.] Université Paris-Saclay, Univ. Paris-Sud, Faculté de Pharmacie, CESP, INSERM UMRS1178, Chatenay-Malabry, 92296, France

[5.] Roche Pharmaceutical Research and Early Development, Therapeutic Modalities, Roche Innovation Center Basel, F. Hoffmann-La Roche Ltd, Grenzacherstrasse 124, 4070 Basel, Switzerland

[#] Equal contributions

[$] Current address: Bridge Pathology Ltd, 637 Gloucester Road, BS7 0BJ, Bristol, UK

[+] Current address: Dept. of Cellular and Molecular Medicine University of California San Diego, School of Medicine, 9500 Gilman Drive La Jolla, CA 92093

[§] Current address: Novartis Institute for Biomedical Research, michael.saxe@novartis.com

Supplementary Information

- Supplementary Materials and Methods
- Supplementary Figures
- Supplementary Table

Supplementary Materials and Methods

Discovery proteomics of neural progenitor cells (NPCs).

NPCs were cultivated in three biological batches using the standardized protocol described below. NPCs were first grown to confluence in an undifferentiated state (approximately 10-15x10^6 cells/T-175). At t = 0, NPCs were seeded in a fresh T-175 at a density of 50,000 cells/cm^2 (approximately 8.7x10^6 cells) and grown in differentiation medium (depleted of FGF and EGF) for the indicated times. The control t = 0 culture was also transferred to a fresh T-175 but was cultivated in presence of growth factors for an additional 24 h before collection. Thus, for each biological replicate, each time point (t = 0, 1, 2, 4, 7, 14, 21 and 28 days) represented a separate cell culture (in a separate T-175 dish) sharing the same biological origin. At each time point, cells were collected from a given T-175 dish, rinsed with PBS, pelleted at low speed and stored at -80°C until processed.

NPCs collected from one T-175 dish (approximately 10-20x10^6 cells) were lysed by sonication in 1 ml of 40 mM Tris and 600 mM NaCl, pH 8.8, supplemented with Protease inhibitor complete (Roche) and 3 μl of Benzonase (Sigma). The lysates were then subjected to ultracentrifugation at 100,000 g for 1 h at 10°C. The insoluble membrane fraction was then washed twice with 1 M KCl and then twice with 0.1 M Na_2CO_3 (each wash followed by a 1 h centrifugation step at 50,000 g at 4°C) to remove the bulk of the contaminating soluble proteins. The crude membrane protein fraction was then solubilized in 8 M urea, 1% (w/v) CHAPS and 50 mM Tris/HCl, pH 8.8, and the total protein concentration was estimated using the Bradford assay (Bio-Rad). After proteins were reduced and alkylated with DTE and iodoacetamide, respectively, 10 μg of total protein was applied onto two duplicate 4-12% Bis-Tris

NuPAGE gels (MES buffer system, Novex), with each gel encompassing one complete biological experiment (one time point per lane). In addition, for each biological experiment, 10 μg of a sample consisting of an equivalent amount of protein pooled from each time point was also analyzed separately on a separate SDS-PAGE gel (2 lanes/biological replicate). After the proteins were stained with Colloidal Blue (Novex), each lane was then gridded into 12 bands each (using the molecular mass markers as a reference) that were subsequently subjected to in-gel digestion as described below. Each sample was destained with 50 mM ammonium bicarbonate and 30% (v/v) acetonitrile and then briefly dried in a Speed-Vac concentrator before being re-suspended in 20 μl of 50 mM ammonium bicarbonate containing 10 ng trypsin (Promega) overnight at room temperature. Peptides were successively extracted using 100 μl of acetonitrile, 100 μl of 1% (v/v) formic acid and 100 μl of acetonitrile, and the three fractions were pooled and dried using a Speed-Vac concentrator. Samples were stored at 4°C until use.

The two technical replicates from each biological experiment were analyzed in 4 batches of 48 samples each. For each batch, the 16 (2 gels x 8 time points) samples belonging to the same molecular mass range were run in succession to increase comparability, whereas the next 16 samples were chosen from a different molecular mass range to avoid carry-over between two neighboring bands (see the analysis scheme in Supplementary Figure 1). The 72 runs that originated from the SDS-PAGE gel with the pooled samples were analyzed in two batches of 36 using a similar analytical scheme.

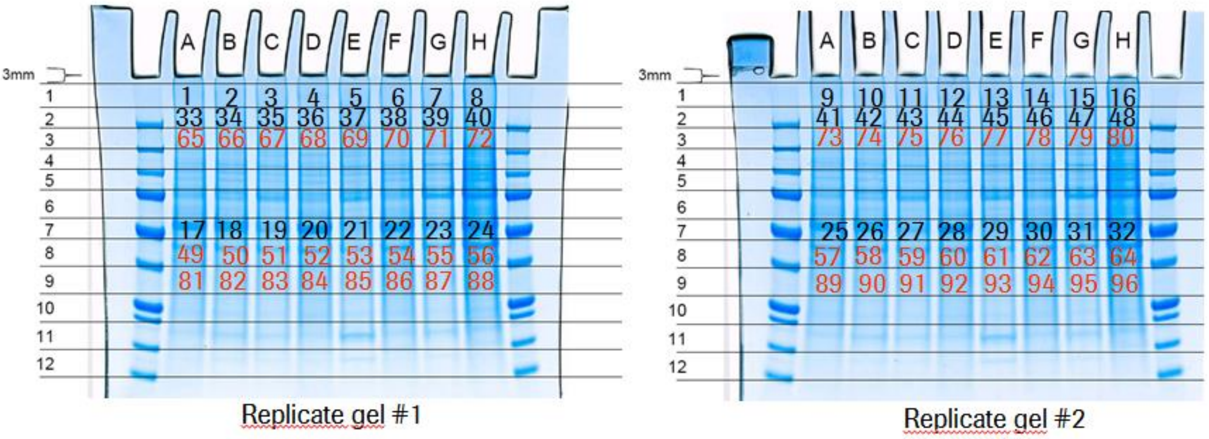

Supplementary Figure 1: SDS-PAGE and LC-MS analysis. Ten micrograms of total protein were applied in duplicate onto 4-12% Bis-Tris NuPAGE gels. After staining with Colloidal Blue, each gel was gridded into 12 fractions, and each sample was collected in individual Eppendorf tubes. Samples were destained and in-gel trypsinized, and peptides were collected into fresh tubes. The LC-MS analysis was performed in the order as indicated on the gel. A: 21-day; B: 7-day; C: 2-day; D: 28-day; E: 0-day; F: 14-day; G: 1-day; and H: 4-day samples.

Peptide samples were analyzed by nanoscale LC-ESI-MS/MS using an Ultimate 3000 nanoflow chromatographic system (Dionex) coupled to a LTQ-Orbitrap tandem mass spectrometer (Thermo Fisher Scientific) equipped with a nanoelectrospray ion source (Proxeon Biosystems). Peptides were separated using an analytical fused silica emitter (75/360 μm i.d./o.d., tip diameter 8±1 μm, New Objective) home-packed with 15-19 cm of 3 μm Reprosil C18 AQ reverse phase material (Dr. Maisch) at 400 bars. Each peptide sample was dissolved in 20 μL of buffer A (2% can and 0.5% acetic acid), from which 5 μL (diluted 1:5 in the same buffer) was loaded at a flow rate of 450 nL/min in 100% buffer A for 12 min. After loading, the flow was decreased to 250 nL/min, and the peptides were eluted from the reverse phase column as follows: 12-14 min, 0-5% buffer B (80% ACN and 0.5% acetic acid); 14-30 min, 5-

30% buffer B using curve 4 (slightly concave) of the Chromeleon software (Dionex); and 30-90 min, 30-55% Buffer B using curve 6 (slightly convex) of the Chromeleon software. The column was then washed with 100% buffer B for 15 min at a rate of 350 nL/min and re-equilibrated in 100% buffer A at a rate of 350 nL/min for 25 min.

Peptides were analyzed by tandem mass spectrometry using the following standard operating parameters: the electrospray voltage was set to 2.0 kV, and the capillary temperature was set to 170°C. Survey scans (scanning range m/z 400-1650) were recorded in the Orbitrap mass analyzer at a resolution of 30,000 with the lock mass option enabled. Data-dependent MS/MS spectra for the five most abundant ions from the survey scan were recorded in the LTQ ion trap using a normalized collision energy of 32% for MS/MS (30 ms activation, q=0.25) and a selection threshold of 500. Target ions selected for MS/MS were dynamically excluded for 30 sec.

Raw data were processed using the SEQUEST search algorithm 27 (SEQUEST version 27.0, revision 12, Thermo Electron). Searches were performed against the UniProtKB/Swiss-Prot protein knowledgebase database (version 2011_07) filtered for “Homo sapiens” (58,772 sequences concatenated with their decoy entries). The data were searched with a mass tolerance of +/- 5 ppm for parent ions and +/- 1.0 Da for the fragment ions. Methionines (reduced/oxidized; +15.9949 Da) were considered differential modifications, and cysteines were considered fully carbamidomethylated (+57.0199 Da). Only fully trypsinized peptides with no more than one missed cleavage were considered for data analysis. The False Discovery Rate (FDR) of the proteins identified in this study was evaluated using the Roche algorithm “Clotho” (1), and only proteins with an FDR ≤1% were examined further. When possible, identifications were reduced to a single entry per protein family by keeping the

canonical UniProtKB/Swiss-Prot naming convention if equivalent isoforms were reported.

(1) Berntenis, N., Kux van Geijtenbeek, S., Ducret, A. A novel protein scoring algorithm for shotgun proteomics. *Annual congress of the Human Proteome Organization (HUPO)* 2009, C522.

Glypican-2 interaction profiling by immunocompetitive capture mass spectrometry

Immunocompetitive capture mass spectrometry was performed essentially as previously described (*1*). Briefly, 21-day differentiated NPCs were harvested and lysed in Dulbecco's phosphate-buffered saline containing $CaCl_2$, $MgCl_2$ (Gibco), 1% NP40 (Calbiochem) and protease inhibitor, complete, EDTA-free (Roche). After a 15-min incubation on ice, lysates were cleared by centrifugation at 14,000 rpm for 10 min at 4°C, and protein concentrations were estimated using the bicinchoninic acid protein assay kit (Pierce). In-house produced anti-GPC2 or anti-FGF2 antibodies (Abcam ab126861) were coupled to Affi-Gel 10 agarose beads (Bio-Rad) for 3 h at 4°C. Unreacted binding sites were blocked by the addition of 0.2 M ethanolamine, and the beads were washed four times with cold PBS and stored at 4°C.

Beads (20 µl) were incubated with cell lysates (850 µg total protein per condition) for 1 h at 4°C, washed four times with lysis buffer and eluted with SDS sample buffer. For the competition experiments, cell lysates were pre-incubated with increasing amounts of free anti-GPC2 antibody (0, 100, 250, 500 and 1,000 ng) in triplicate for 1 h before incubation with the immobilized anti-GPC2 antibody. Eluates were separated on a 4–20% Tris-Glycine SDS-PAGE gel, and proteins were stained with Colloidal Blue (Novex). Four bands ranging from 20 to 120 kDa were excised and subjected to in-gel digestion with trypsin using standard procedures.

Samples were analyzed with a nanoflow Easy-nLC system (Proxeon) connected to an LTQ-Orbitrap Velos (Thermo Fischer Scientific). Peptides were loaded onto an AQUA C18 trap (100 μm x 10 mm, Phenomenex) and separated on a ReproSil-PurC18-AQ (75 μm x 200 mm, 3 μm particle size, 120 Å, Dr. Maisch GmbH) analytical column using a 50-min gradient of 0-35% acetonitrile (with 0.6% acetic acid) at a rate of 250 nL/min. Full-scan MS spectra were acquired in the Orbitrap with a resolution of r = 60,000 (at m/z 400). The detected ions were recalibrated on the fly using the ambient air polysiloxane at m/z 445.120024 as the lock-mass. The 10 most intense ions with a charge state >1+ were selected for collision-induced dissociation in the linear ion trap and then excluded for 30 s. Raw MS files were converted into .dta files using Extract-MSn (version 1.0.0.8) and searched against a database consisting of the human part of the SwissProt database (August 2011, 35 106 entries, including splice variants) using Sequest (version 27.0, revision 12, both Thermo Fisher Scientific). Only fully trypsinized peptides with no more than one missed cleavage were considered, and mass tolerances of 5 ppm and 10 ppm were used for the precursor and fragment ions, respectively. Oxidized methionine (+15.9949 Da) was set as a differential modification, and carbamidomethylated cysteines (+57.0215 Da) were set as a static modification. The spectral false discovery rate (specFDR) was restricted to 1.0% by performing a target-decoy search using a concatenated-decoy database.

The top ranked GPC2-interacting protein candidates FGF2, ECHA and ECHB were confirmed by Western blotting. The proteins were detected with Abcam anti-ECHA (ab54477), anti-ECHB (ab88256) and anti-FGF2 (ab126861) antibodies; the latter was also used for the co-immunoprecipitation experiment.

Supplementary Figures

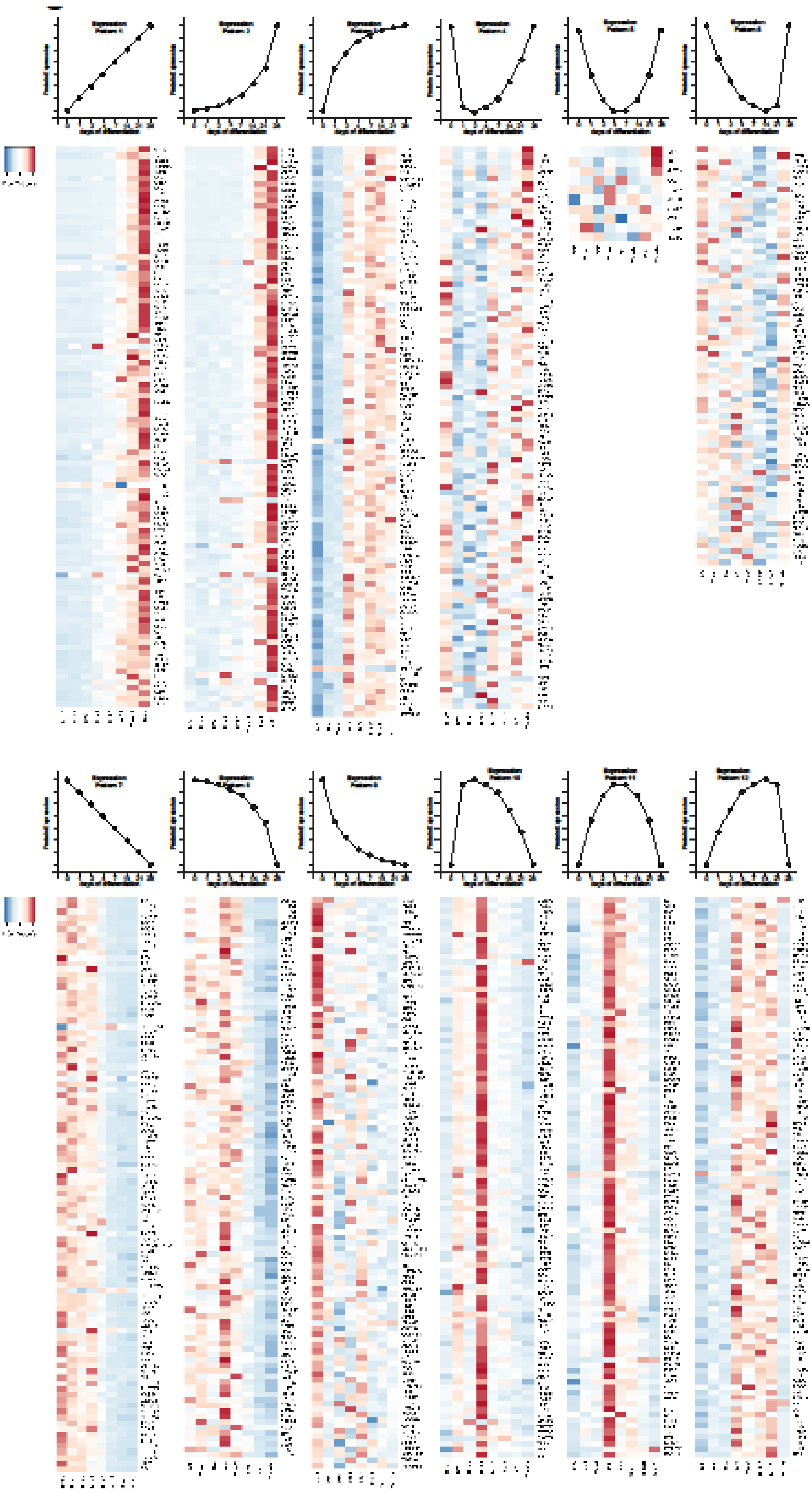

Supplementary Figure 1: Cluster analysis of proteins expressed during neuronal differentiation. The protein peptides were clustered into expression patterns (1-12) according to their mode of expression. The expression of proteins in clusters 1-3 increased during neuronal differentiation; thus, these proteins likely had a neuronal function. Clusters 7-9 displayed the highest expression in undifferentiated neural progenitor cells. Clusters 4-6 and clusters 10-12 showed the lowest/highest expression, respectively, at intermediate time points during differentiation.

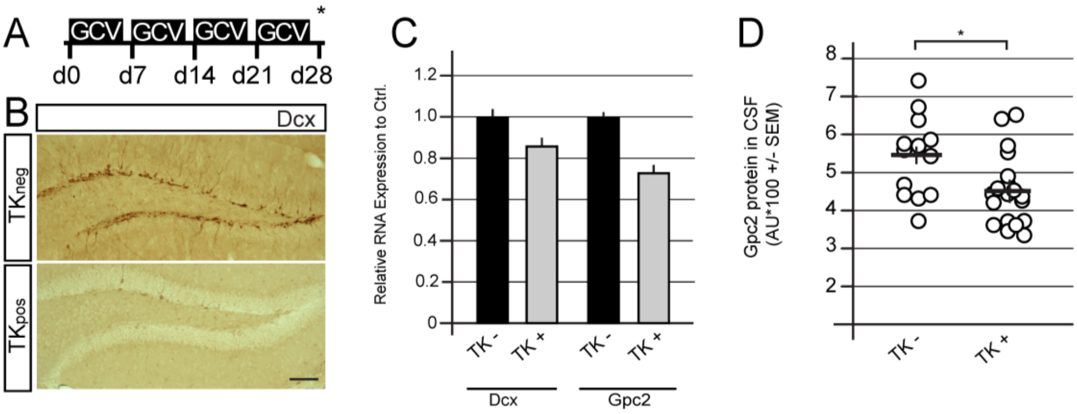

Supplementary Figure 2: GFAP-TK-mediated ablation of neurogenesis.

A) Experimental paradigm for GFAP-TK-mediated ablation of neurogenesis. Animals were fed chow containing Ganciclovir (GCV) 5 days per week for 4 weeks to induce targeted ablation of the $GFAP^+$ neural stem cells.

B) Representative images of Dcx immunostaining.

C) Relative expression of Dcx and Gpc2 mRNAs in micro-dissected hippocampi from the TK^- and TK^+ animals. The ablation of neurogenesis (TK^+) reduced the expression of Dcx and Gpc2.

D) Gpc2 levels in the CSF in response to the GFAP-TK-mediated loss of neurogenesis. The loss of neurogenesis led to a significant reduction in the CSF levels of Gpc2.

Error bars shown in C represent the SEM. Error bars shown in B represent CI. Scale bar = 100 μm. $p<0.05$ = *; $p<0.01$ = **; $p<0.001$ = ***.

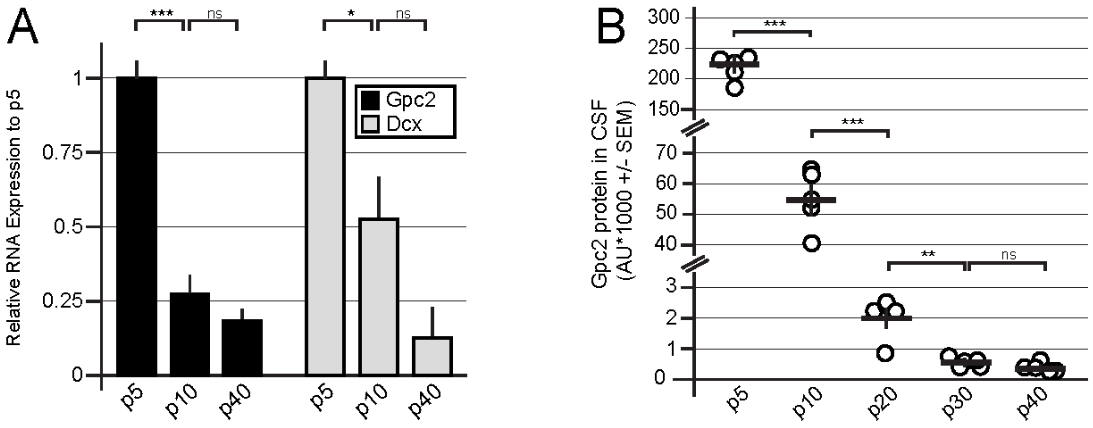

Supplementary Figure 3: Glypican- 2 expression strongly decreases during postnatal development.

A) Expression of Gpc2 and Dcx mRNAs in early postnatal rats. The expression of both Gpc2 and Dcx is substantially decreased within the early postnatal period, concomitant with the reduction in neurogenesis.

B) Gpc2 levels in rat CSF decrease during the early postnatal period.

The error bars shown in B represent the SEM. The error bars shown in A represent CI.

Scale bar = 200 μm. p<0.05 = *; p<0.01 = **; p<0.001 = ***.

A

Protein Name	Abbreviation	Signal Reduction (%)	p-value	Competitor antibody (μg/ml +/- SEM) 0	1	2.5	5	10
Glypican 2	GPC2	91.66	0.000682	17.32 +/- 0.11	17.33 +/- 0.07	16.82 +/- 0.14	15.11 +/- 0.04	13.71 +/- 0.11
Trifunctional enzyme subunit β	ECHB	90.43	0.000582	17.20 +/- 0.12	17.13 +/- 0.15	16.81 +/- 0.17	15.46 +/- 0.07	13.91 +/- 0.08
Trifunctional enzyme subunit α	ECHA	89.86	0.000686	17.07 +/- 0.06	16.97 +/- 0.06	16.45 +/- 0.13	15.51 +/- 0.01	13.84 +/- 0.11
Fibroblast growth factor	FGF2	89.48	0.000898	15.12 +/- 0.54	15.22 +/- 0.30	15.10 +/- 0.01	11.43 +/- 0.22	12.31 +/- 087
Cysteine-rich fibroblast growth factor receptor	GSLG1	79.38	0.000971	15.94 +/- 0.13	15.53 +/- 0.08	14.91 +/- 0.03	13.60 +/- 0.09	13.71 +/- 0.21
Sequestome-1	SQSTM	78.6	0.000593	15.76 +/- 0.37	15.86 +/- 0.27	15.62 +/- 0.16	14.25 +/- 0.47	13.49 +/- 0.28
Protein acyl-coenzyme A thioesterase 8	ACOT8	76.75	0.000586	15.68 +/- 0.12	15.78 +/- 0.20	15.13 +/- 0.14	13.94 +/- 0.22	13.51 +/- 0.11
Hypermethylated in cancer 2	HIC2	74.35	0.000707	15-37 +/- 0.19	15.79 +/- 0.20	15.26 +/- 0.23	14.11 +/- 0.84	13.40 +/- 0.30
Leucine-rich PPR motif-containing protein	LPPRC	74.33	0.000991	14.75 +/- 0.17	14.34 +/- 0.09	14.18 +/- 0.25	12.87 +/- 0.01	13.05 +/- 0.15
Sphingosine-1-1phosphate lyase 1	SGPL1	68.41	0.00059	15.25 +/- 0.13	14.88 +/- 0.33	14.32 +/- 0.27	13.50 +/- 0.07	13.35 +/- 0.19
General transcription factor 3C polypeptide 1	TF3C1	60.19	0.001001	14.76 +/- 0.08	14.95 +/- 0.13	14.30 +/- 0.17	13.09 +/- 0.12	13.40 +/- 0.32
				Rel. Protein abundance (mean +/- SEM)				

B

Experiment	Figure	Gpc2 in CSF (Mean$_{Arbitrary\ Units}$ x 100 (+/- SEM))							n
Postnatal development (rat)	3	p5	p10	p20	p30		p40		5/group
		2245.26 (+/- 106.81)	553.79 (+/- 44.72)	20.17 (+/- 2.75)	5.57 (+/- 1.04)		3.91 (+/- 1.11)		
Postnatal development (mouse)	3	w4	w13		w24		w42		min. 6/group
		8.84 (+/- 0.1245)	8.113 (+/- 0.5202)		5.972 (+/- 0.3957)		4.628 (+/- 0.2523)		
Physical Exercise	4	Ctrl			Run				10/group
		7.776 (+/- 0.2779)			9.642 (+/- 0.2726)				
Aging	6	young			aged				min. 10/group
		5.111 (+/- 0.3088)			3.413 (+/- 0.2457)				
Loss of neurogenesis (Rbpj deletion)	3	Ctrl (Rbpj$^{+/+}$)			Mutant (Rbpj$^{-/-}$)				6/group
		6.245 (+/- 0.442)			4.418 (+/- 0.4098)				
Loss of neurogenesis (GFAP-Tk)	SF2	Ctrl (GFAP-TK-)			Mutant (GFAP-TK+)				min. 14/group
		5.454 (+/- 0.276)			4.539 (+/- 0.2175)				
Human age groups	6	20s	30s	40s	50s	60s	70s	80s	min. 5 / max. 22 per age group
		4.181 (+/- 0.6218)	6.271 (+/- 0.8356)	8.567 (+/- 0.646)	4.529 (+/- 0.8982)	5.093 (+/- 0.7387)	7.414 (+/- 0.1496)	7.059 (+/- 0.1877)	

Supplementary Table:

A) Top 10 proteins with an ICC-MS displacement profile.

B*)* Results of the Gpc2 measurements in CSF.
